# Application of Convolutional Neural Networks for Automated Ulcer Detection in Wireless Capsule Endoscopy Images

**DOI:** 10.3390/s19061265

**Published:** 2019-03-13

**Authors:** Haya Alaskar, Abir Hussain, Nourah Al-Aseem, Panos Liatsis, Dhiya Al-Jumeily

**Affiliations:** 1 Department of Computer Science, College of Computer Engineering and Sciences Prince Sattam Bin Abdulaziz University, Alkharj 11942, Saudi Arabia; N.alaseem@psau.edu.sa; 2 Department of Computer Science, Liverpool John Moores University, Liverpool L3 3AF, UK; a.hussain@ljmu.ac.uk (A.H.); D.AlJumeily@ljmu.ac.uk (D.A.-J.); 3 Department of Computer Science, Khalifa University of Science and Technology, Abu Dhabi 127788, UAE; panos.liatsis@ku.ac.ae

**Keywords:** deep learning networks, AlexNet, GoogLeNet, convolutional neural networks, wireless capsule endoscopy, ulcer detection

## Abstract

Detection of abnormalities in wireless capsule endoscopy (WCE) images is a challenging task. Typically, these images suffer from low contrast, complex background, variations in lesion shape and color, which affect the accuracy of their segmentation and subsequent classification. This research proposes an automated system for detection and classification of ulcers in WCE images, based on state-of-the-art deep learning networks. Deep learning techniques, and in particular, convolutional neural networks (CNNs), have recently become popular in the analysis and recognition of medical images. The medical image datasets used in this study were obtained from WCE video frames. In this work, two milestone CNN architectures, namely the AlexNet and the GoogLeNet are extensively evaluated in object classification into ulcer or non-ulcer. Furthermore, we examine and analyze the images identified as containing ulcer objects to evaluate the efficiency of the utilized CNNs. Extensive experiments show that CNNs deliver superior performance, surpassing traditional machine learning methods by large margins, which supports their effectiveness as automated diagnosis tools.

## 1. Introduction

Given that deep learning tools were successfully applied to image analysis, researchers have explored their application in medical image analysis [1,2,3,4,5,6,7,8]. Deep learning has proven to be a powerful machine learning tool and has demonstrated its ability in automated diagnosis of diseases [2,3,9]. Therefore, it has been considered for use in medical image analysis and recognition. It can improve medical image examination by enhancing the abilities of clinicians and health professionals in the context of early diagnosis. Consequently, it can potentially help in prognosis and in the development of effective disease treatment regimes.

In medical image applications, several deep learning models were developed and applied [3,4,6,9]. One type of high-performance deep learning network is convolutional neural networks (CNNs), which demonstrated a crucial capacity to automatically extract high-level features from multi-dimensional data, whilst exhibiting high accuracy rates. CNNs are able to process data in various forms, e.g., multi-dimensional features, including signals, images, and videos [7,8,10,11]. The architecture of CNN is designed as a series of layers, particularly, convolutional layers and pooling layers, followed by fully connected layers.

A review of the literature shows that CNNs were successfully used to detect, segment and recognize objects and regions in images [5,9,12,13,14,15]. Litjens et al. [9] conducted a survey of medical image analysis using deep learning algorithms. This indicated that deep learning has covered almost every aspect of medical image analysis. In addition, the work noted that many pre-trained networks were used as feature extractors and that various CNN architectures were chosen to segment and classify a variety of medical images. Conversely, Szegedy et al. [12] focused on an efficient deep neural network architecture for computer vision. In [16], CNN models were used to detect polyps in colonoscopy videos. Each CNN model used individual features, including texture-, shape-, and color-based ones, which were combined with temporal information to detect the occurrence of polyps. Next, the results of these individual models were fused together and final decisions were made. Finally, Wolterink et al. [8] used paired CNNs to detect coronary calcium in CT angiography images. Motivated by the performance of deep neural networks in medical image analysis, researchers have aimed to further enhance the performance of deep neural networks by fine tuning their network configuration by investigating different types of architectures, varying numbers of layers, learning rates, etc. The aim of the present study is to investigate the use of deep learning networks in the context of ulcer detection in WCE images with high accuracy and speed. Owing to the limited size of available data, pre-trained CNN architectures were considered, as recommended in [17].

Along this direction, the present study attempts to examine for the first time the performance of two pre-trained CNNs, GoogLeNet [12], and AlexNet [13], in terms of their ability to recognize abnormalities, i.e., the occurrence of ulcers, in WCE images. In particular, the CNNs are trained on WCE images to detect and classify ulcers, which are known to be the most common gastrointestinal (GI) abnormality [18,19]. Furthermore, the potential of these two CNNs for ulcer detection is validated using a dataset consisting of 526 images taken from WCE videos, using a variety of performance criteria, including sensitivity, specificity, accuracy, loss, and area under curve (AUC).

The remainder of this paper is organized as follows. Wireless Capsule Endoscopy (WCE), which was applied to validate the architecture of the two CNNs is explained and related works are briefly reviewed in Section 2. Then, a concise literature review in the subject area of the research is presented in Section 3. The present study’s dataset and methods are described in Section 4. Next, the experimental results are presented and discussed in Section 5. Finally, the conclusions of this work and suggestions for future research are presented in Section 6.

## 2. Wireless Capsule Endoscopy Image Ulcer Detection Techniques

In this section, we provide an overview of wireless capsule endoscopy (WCE) and the use of deep learning for the analysis of WCE images.

### 2.1. Wireless Capsule Endoscopy

Compared to traditional endoscopy, WCE provides low-risk, noninvasive image-video inspections of patients’ digestive tracts [6]. WCE has been considered a first-line examination tool that detects abnormalities, including Crohn’s disease, ulcers, bleeding and polyps [9], during routine checks. WCE can provide useful insights for several types of ulcers affecting the GI tract, including esophageal [20], peptic [19], gastric [21], and duodenal [22]. For each patient, more than 55,000 images of the GI tract are captured, but evidence of abnormalities may appear in only a few of them. Therefore, physicians often spend hours analyzing the images [23], which may involve various challenges, including low contrast and complex background, variations in lesion shape and color, thus affecting the accuracy of segmentation and subsequent classification [24]. These issues complicate objective disease diagnosis, thus necessitating the opinions of multiple experts to avoid misdiagnosis [25]. Consequently, there is high demand for an alternative method for automated detection of GI abnormalities, and considerable effort has been directed into the automatic inspection and analysis of WCE data.

The majority of works in the literature focus on the analysis of textural features, extracted from WCE images. Studies used traditional machine learning techniques to detect abnormalities in images containing texture-based features [26,27,28,29,30]. Li and Meng [25,27] proposed curvelet-based local binary patterns (LBP) as features to detect ulcer regions, as they capture multi-directional features and show robustness to illumination changes. Both multi-layer perceptrons (MLP) and support vector machines (SVM) was used in ulcer region recognition. MLP had the highest accuracy, i.e., 93.28%, while SVM achieved 88% sensitivity [27]. Along the same direction, local binary pattern variance and the discrete wavelet transform were used in [26] to automatically detect abnormalities in WCE images. Computer-aided detection of ulcers in WCE images was pursued in [28] using an approach based on completed LBP and Laplacian pyramid. It was found that the magnitude of the proposed descriptor is robust against illumination changes. Moreover, they evaluated detection accuracy in the green and Cr components in RGB and YCbCr color spaces, achieving average accuracies of 95.11% and 93.88%, respectively, using support vector machines (SVM). In [29], a large number of features based on both texture and color information were considered in the context of GI abnormality detection and segmentation using a variety of machine learning paradigms. Experimental results indicated that the SVM combined with sequential floating forward search (SFFS) and the proposed vector supported convex hull (VSCH) algorithms performed best in detection of bleeding, focal, and excessive ulcers. Sources of noise, such as air bubbles, are first addressed in [30] the color-saliency region detection (CSD) method, followed by feature extraction using the color channels modelling of local binary pattern operator (CCLBP). SVM is used for detection of the lesions of interest. In this method, CCLBP combines both color and grayscale information, thus providing robustness to illumination changes, while maintaining highly discriminative features for classification purposes. Bchir et al. [31] evaluated nine visual features, including local binary patterns, CIE lab color histograms, curvelet transforms, chromaticity moments color, scalable color descriptors, color coherence vectors, homogeneous texture descriptors, YCbCr color histograms, and HSV (hue, saturation, and intensity value) color histograms for ulcer detection in WCE video frames. They reported 96% accuracy when using SVM.

### 2.2. Deep Learning Network in WCE

The evolution of deep learning provided new opportunities to improve the analysis of WCE images [10,32,33,34,35]. A review of the literature shows that deep learning has proved to be more successful than traditional machine learning tools. In 2018, Fan et al. [10] used deep learning to analyze WCE frames in detection of both ulcers and erosions that appear in the intestine. The study applied the AlexNet CNN and achieved high accuracy (95.16%) and sensitivity (96.80%), demonstrating the efficiency of the deep learning approach. Our proposed methodology is different to [10] in which we utilized AlexNet using different WCE data sets and learning parameters.

Jia et al. [33] used deep CNN to automatically detect bleeding in WCE images. Their experiment achieved a F-measure score of 99%. Pei et al. [35] have also used fully convolutional networks (FCNs) with long short-term memory (LSTM). The FCN-LSTM was trained on five cine MRI sequences that had no labeling, and the FCN network was trained on a large dataset of 50 raw cine MRI sequences, which were labeled.

Segu et al. [35] used a computer aided decision system based on a generic feature-learning approach for WCE. They built a large dataset of 120,000 labeled WCE images to train the CNN. Their results achieved a classification accuracy of 96%. Wimmer et al. [34] trained a CNN using different numbers of layers and filters and various filter dimensions in order to diagnose celiac disease. They combined CNN with SVM. The highest performance achieved was 97%.

In [32], automated feature extraction using a CNN architecture was applied, and then the extracted features were used to train SVM to detect inflammatory gastrointestinal disease in WCE videos. The study achieved accuracies of up to 90%. Yuan and Meng [14] proposed a deep learning network named stacked sparse auto-encoder with image manifold (SSAEIM). It was used to recognize polyps in WCE images. The study found that the SSAEIM was able to detect polyps, bubbles, and turbid images with 98.00, 99.50, and 99.00% accuracy, respectively.

## 3. Literature Review

Traditional endoscopy techniques used for the detection of ulcer are invasive and painful procedures. To overcome these limitations, wireless capsule endoscopy examinations are introduced to provide non-invasive, painless, and effective diagnosis of the gastrointestinal tract [26].

Yuan et al. [36] proposed a two-stage, fully automated computer-aided detection system for the detection of ulcers in WCE images. First, they automatically detect salient regions across WCE images and overcome problems associated with the use of traditional methods, which ignore neighboring and boundary information of the object. Then, they use the locality-constrained linear coding (LLC) method for the classification of ulcers.

In [37], the authors proposed the use of color features—including RGB, HSV, and CCV—to analyze the status of the small intestine. Their extensive experiments indicated that the C4.5 method generated the best results for the classification of bleeding and ulcers in WCE images. Furthermore, their investigations showed that CCV features enhanced the efficiency of WCE image analysis.

Nawarathna et al. [38] combined the Leung and Malik (LM) filter and LBP to propose a new method for detection of abnormalities in endoscopy videos. The authors used the KNN algorithm to classify image blocks, based on the distribution of textures.

In contrast to previous researches, this work proposes the novel application of pre-trained convolutional neural network, GoogleLeNet, for the detection of ulcers in WCE images. While the use of machine learning algorithms, such as SVM [31] and KNN [38], for the classification of WCE image necessitates the use of extensive and time-consuming feature selection techniques, the proposed methodology using CNN automatically extracts features from WCE images and successfully detects ulcers. To demonstrate the benefits of the CNN approach, we perform extensive simulations using a number of features extracted from WCE images, namely, color histograms, LBP and color coherence, and MLP networks to evaluate the quality of feature information and its effect on classification accuracy. Moreover, we provide performance comparisons with state-of-the-art machine learning algorithms.

## 4. Methods

### 4.1. Convolutional Neural Networks

The architecture of CNN is different from that of regular neural networks. CNN layers have neurons organized in three dimensions, namely, width, height, and depth, where every layer in a CNN converts a 3D input volume into a 3D output volume of neuron activations. Typically, there are three types of layers in a CNN architecture, i.e., convolutional, pooling, and fully connected layers. It is not necessary that all neurons in one layer are connected to all the neurons in the next layer. Sequences of convolutions and pooling processes are performed on the input data with the use of a filter to produce a feature map. These feature maps are combined together as the final output of the convolution layer.

The convolutional layer is considered the essential block of a CNN and correspondingly makes training of CNN time consuming. In these layers, a convolution operation is applied to the input in order to compute the outputs of neurons. The parameters of convolutional layers are shared sets of weights (also known as kernels or filters), which have very small receptive fields.

Pooling layers employ nonlinear down sampling procedures. Max pooling is a popular nonlinear operation. Here, the input is divided into a group of non-overlapping frames and the maximum for each group is the output. In this way, max-pooling layers reduce the number of parameters, the possibility of overfitting, and the computational complexity of the network. Therefore, a max-pooling layer is usually inserted between convolutional layers.

Dropout layers are usually inserted to reduce the risk of overfitting. The main role of the dropout layer is to drop neurons in the CNN and their connections with a certain probability [39]. The most common activation function is the non-saturating ReLU (rectified linear unit). Fully connected layers perform as a classifier with all neurons in a fully connected layer being fully connected to all outputs of the previous layer [9]. However, it is worth mentioning that training CNN from scratch requires large amounts of training data, which is not always available and can cause overfitting. Therefore, in this study, pretrained CNN with appropriate fine-tuning will be utilized, as described in Section 4.2.

The CNN was implemented by using two popular types of pretrained CNN architectures, namely, GoogLeNet [12], and AlexNet [13].

### 4.2. Pretrained Networks

A pretrained network has pretrained weights, which can be used in a related task. Chen et al., asserted that in order to use CNNs in domains where the limitation is the size of the dataset, pretrained network may be the solution [40]. Additionally, training CNN from scratch is time-consuming and needs an extensive amount of computational power and memory capacity. Numerous studies considered using pretrained CNN and asserted that this type of CNN can improve accuracy in the case of limited datasets [17,41,42]. Yosinski et al. [42] claimed that weights from a distant task may achieve better performance than using randomly initialized weights.

In the literature, several pretrained CNN exist such as AlexNet, VGGNet, GoogLeNet, and ResNet. However, GoogLeNet and AlexNet are usually applied for feature extraction and classification and yield very good results. For example, they have been used in medical data analysis, including anatomical applications [7,16,43], computed tomography [44], biomedical signal processing [45,46], e.g., interstitial lung disease [6], GoogLeNet and AlexNet were also used in recognition of malaria-infected cells, where GoogLeNet and AlexNet achieved 98.13% and 95.79% accuracy, respectively. Traditional machine learning tools, including SVM, obtained an accuracy of 91.66% [4]. Inspired by the superior performance of these two CNNs, the current research investigates the best configuration of these two widely used CNNs for ulcer detection and classification.

The pretrained networks were fine-tuned by freezing the weights of the first layers—i.e., the weights of the frozen layers were not adjusted during system training—whereas the fully connected layers, responsible for mapping the feature representations extracted by the initial layers into the class label information, were fine-tuned.

All the weights in the fully connected layers were initialized with random values and trained using the stochastic gradient descent (SGD) algorithm.

**Figure 1:**
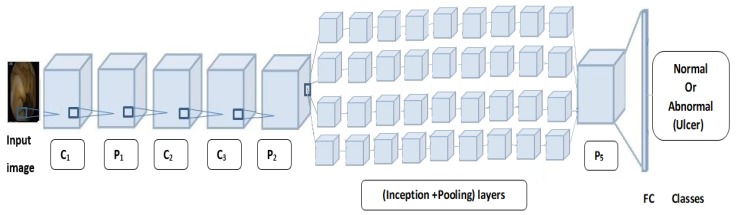
GoogLeNet architecture. Symbols C_i_ refer to convolution layers, P_i_ refer to pooling layers and FC_i_ refer to fully connected layers.

**GoogLeNet:** In 2014, GoogLeNet was the winner of the ImageNet Large-scale Visual Recognition Challenge (ILSVRC), an annual competition that measures developments in object recognition and classification [12]. GoogLeNet achieved an error rate of 6.7%, when used with inception modules, which have various sizes of convolution layers. Figure 1 shows the inception modules used to build the network.

With GoogLeNet, each layer works as a filter. This configuration enhances the abilities of GoogLeNet in detecting the best features in images. The first layers detect common features, including blobs, edges, and colors. The last layers detect high-level features.

In this work, GoogLeNet was retrained to recognize ulcer images by adding four new layers to its structure, specifically, a dropout layer with a 50% probability of dropout, a fully connected layer, a softmax layer and a classification-output layer. The number of outputs of the fully connected layer was set to 2, corresponding to the classes of normal and abnormal (i.e., ulcer). Figure 1 illustrates the layers of GoogLeNet. In the experiments, a total of 144 layers were used to build GoogLeNet.

**Figure 2:**
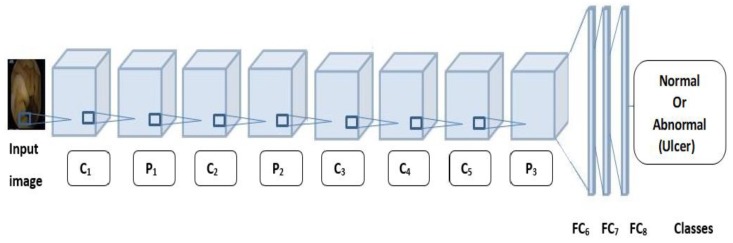
AlexNet architecture with five convolutional and max pooling layers and three fully connected layers for ulcer image detection.

**AlexNet:** Alex Krizhevsky et al. [13] designed a large, deep convolutional neural network, known as AlexNet. The network has 11 × 11, 5 × 5, 3 × 3, convolution, max pooling, dropout, and fully connected layers, as illustrated in Figure 2. There are ReLU activation functions after every convolutional and fully connected layer. The dropout layer has a 50% probability of dropout. The first layers act as feature extractors to determine the high-level features. AlexNet has 25 layers, which is fewer than those in GoogLeNet.

To address the problem of ulcer detection, AlexNet required modification. One of the fully connected layers, Layer 23, was modified to have the same size as the number of classes and the classification output layer, Layer 25, contained the name of the loss function that was used to train the network. Let X be a set of WEC images, let S_sen, S_Sp, S_ac, and S_AUC be elements of the sets of sensitivity, specificity, accuracy, and area under the curve, respectively. The proposed methodology is depicted in Algorithm 1.
**Algorithm 1.**∀ x ∈X, ∃ i ∈ X: i resize of x Let G be GoogLeNet a pretrained network ∈CNN Let A be AlexNet a pretrained network ∈CNN Let M be a set of measures: M = {Accuracy, Sensitivity, Specificity, Loss, AUC} ∀ G & A∈CNN, ∃ m | m [Sensitivity] = {S_sen: S_sen ⇒CNN(x)} & m[Specificity] = {S_sp: S_sp ⇒CNN(x)} & m[Accuracy]= {S_ac: S_ac⇒CNN(x)} & m[AreaUnderCurve] = {S_AUC: S_AUC ⇒CNN(x)}

## 5. Experiments and Results

**Figure 3:**
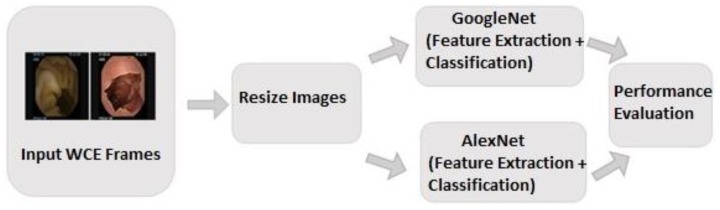
Schematic diagram of the overall system for ulcer detection.

This section describes the experiments carried out to evaluate the two types of CNN in ulcer detection using WCE images. The process is illustrated in Figure 3. The performance of each CNN was evaluated using six types of evaluation metrics: sensitivity, specificity, accuracy, loss, time cost, and area under curve (AUC). The first two metrics were computed by finding the number of true positives, true negatives, false positives, and false negatives. True positives (TP) are correctly detected abnormalities (ulcers). True negatives (TN) are correctly detected normalities (non-ulcers). False positives (FP) and false negatives (FN) are the numbers of incorrect detections of normalities and abnormalities, respectively. The AUC was used to evaluate the performance of the CNN and showed the probability of correctly identified positive instances, which were abnormal instances with higher identification than randomly chosen negative instances, the normality instance in this case.

The five metrics are computed as

Sensitivity = TP/(TP + FN)(1)

Specificity = TN/(TN + FP)(2)

Accuracy = (TP + TN)/(TP + TN + FP + FN)(3)

(4) $$\mathrm{Loss}\;=-\Sigma \;y_{j}\;\log\;({\hat{y}}_{i})\;\left(y\;\mathrm{is}\;\mathrm{the}\;\mathrm{true}\;\mathrm{value}\;\mathrm{and}\;{\hat{y}}_{i}\;\mathrm{is}\;\mathrm{the}\;\mathrm{predicted}\;\mathrm{value}\right)$$

AUC = 0.5 (Sensitivity + Specificity)(5)

The training options were also modified. The size of the mini-batch, which is a subset of the training set to be used in each iteration of the experiment, was set at 20. The maximum number of epochs for training was set at 10. Three learning rate values were explored, i.e., [0.01, 0.001, 0.0001], in order to evaluate the most appropriate setting. The root mean square propagation was used as an optimizer for both CNN networks utilized in the experiments.

The simulations were ran using MATLAB 2018. For the purposes of reproducibility, the networks were trained in a standalone system with an Intel Core Processor i7-7500U CPU at 2.70 GHz, 2904 MHz, 2 cores, and 64 GB of RAM.

### 5.1. Dataset

**Figure 4:**
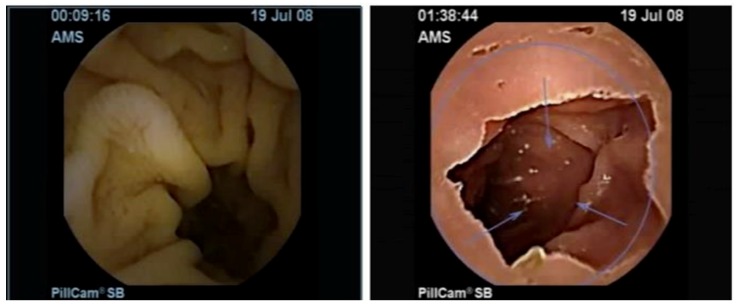
Sample ulcer images from WCE videos. (**Left**) esophageal ulcer; (**Right**) gastric ulcer.

The images used in the experiments were taken from [47]. The dataset consists of 1875 images captured using WCE video, which included 1525 instances of ulcers and 250 instances of the normal class. These images were recorded from two parts of the digestive system, i.e., esophageal and gastric. Figure 4 shows sample ulcer images.

The first step in analyzing the WCE images was dividing them randomly into training and testing sets. 80% of the images, i.e., 421 images, were used for training, and the rest, i.e., 105 images, were used for testing as recommended in [44]. The training set contained 256 abnormal and 80 normal images, while the test set contained 80 abnormal and 25 normal images.

The original images had a resolution of 256 × 256 × 3 (256 width, 256 height, 3 color channels). To fit GoogLeNet, the images were resized to 224 × 224 × 3 pixels, wherein for AlexNet, the images are resized to 227 × 227 × 3 pixels.

### 5.2. Results

**Figure 5:**
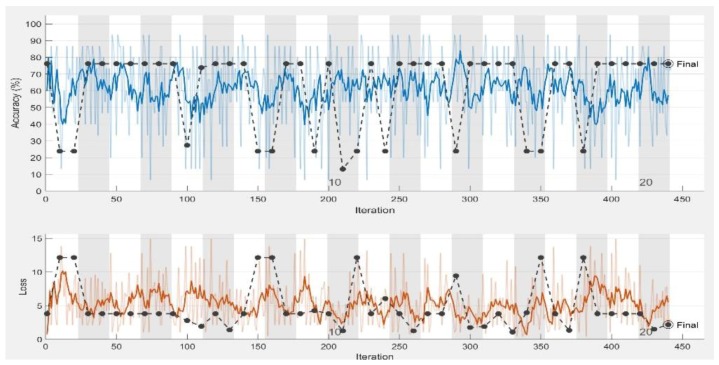
GoogLeNet training process with learning rate of 0.01. The figure shows the epoch number, training accuracy (blue line), validation accuracy (black line), and loss function value for the training data (red line) and validation data (black line).

**Figure 6:**
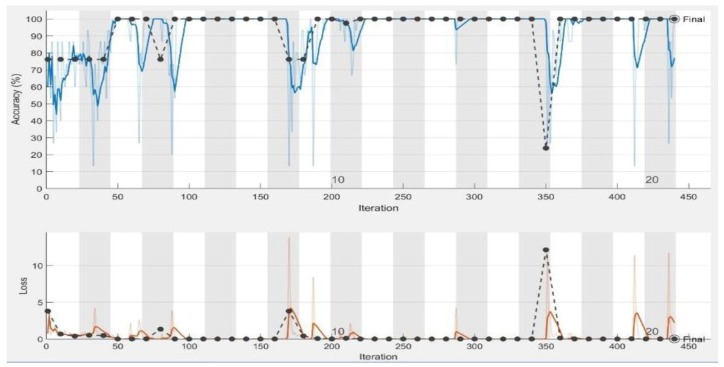
GoogLeNet training process with learning rate of 0.001. The figure shows the epoch number, training accuracy (blue line), validation accuracy (black line), and loss function value for the training data (red line) and validation data (black line).

**Figure 7:**
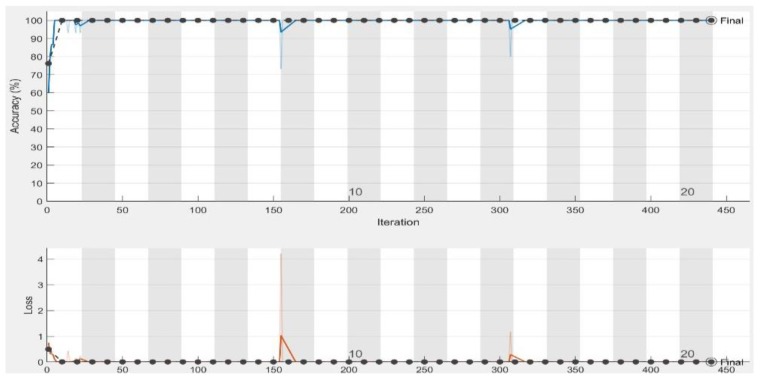
GoogLeNet training process with learning rate of 0.0001. The figure shows the epoch number, training accuracy (blue line), validation accuracy (black line), and loss function value for the training data (red line) and validation data (black line).

**Figure 8:**
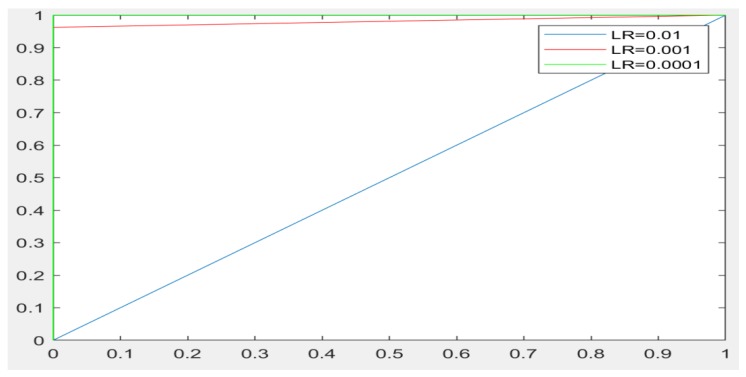
The ROC curves showing the performance of GoogLeNet on testing data sets with learning rates of 0.01, 0.001, and 0.0001.

**Figure 9:**
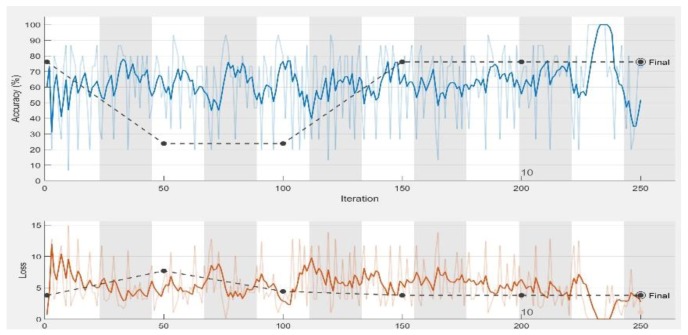
AlexNet training process with a learning rate of 0.01. The figure shows the epoch number, training accuracy (blue line), validation accuracy (black line), and loss function value for the training data (red line) and validation data (black line).

**Figure 10:**
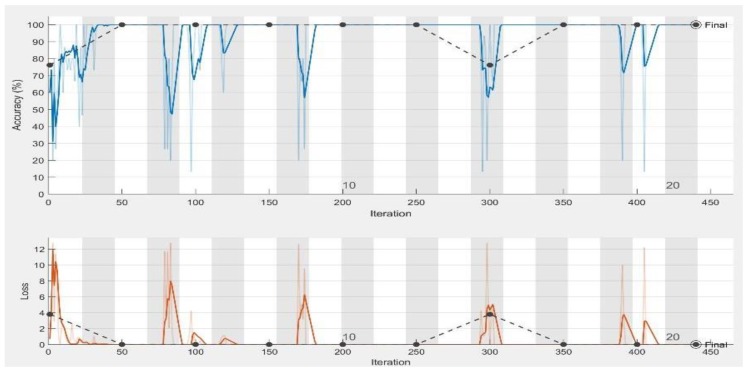
AlexNet training process with a learning rate of 0.001. The figure shows the epoch number, training accuracy (blue line), validation accuracy (black line), and loss function value for the training data (red line) and validation data (black line).

**Figure 11:**
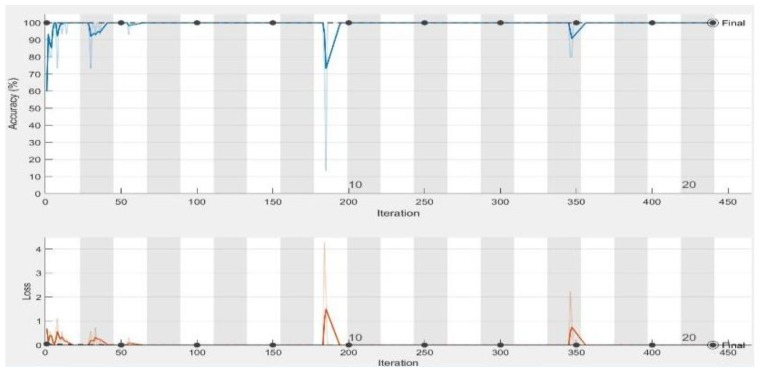
AlexNet training process with a learning rate of 0.0001. The figure shows the epoch number, training accuracy (blue line), validation accuracy (black line), and loss function value for the training data (red line) and validation data (black line).

**Figure 12:**
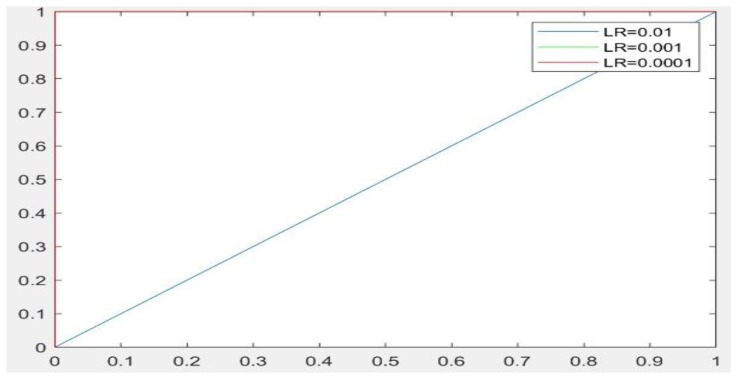
ROC curves showing the performance of AlexNet on testing data sets with the three learning rates of 0.01, 0.001, and 0.0001.

In the experiments, CNN receive an input image, process it and categorize it in two categories (i.e., normal, abnormal (ulcer)). Table 1 and Table 2 summarize the evaluations of GoogLeNet and AlexNet, in the classification of WCE images. Table 3 shows the run times for the pretrained CNN classifiers used in these experiments. Figure 5, Figure 6, Figure 7, Figure 8, Figure 9, Figure 10, Figure 11 and Figure 12 illustrate the performance of GoogLeNet and AlexNet, respectively. Both figures show the training progress over the epochs for both CNN.

From Table 1, it can be observed that the performance of GoogLeNet with 0.01 learning rate achieved less than 77% accuracy. It can be noticed that the lower results are obtained with learning rates of 0.01 and 0.001. It further shows that the networks are not able to successfully detect classes based on the sensitivity and specificity metrics in Table 2, while the performance of the network increases, when the learning rate decreases. Therefore, it is shown that loss is not mitigated when the learning rate increases. If a very small learning rate is selected, the loss function starts reducing in the first few iterations, as illustrated in Figure 7. This shows that the samples of each class are detected correctly, based on the results of Table 1. As it can be seen, the sensitivity and specificity were both 100%. Figure 7 shows that the network is stopped after 440 iterations. As shown in the first epoch, the network achieved 100% accuracy, after which it was unstable until epoch 14. Then, it obtained 100% accuracy and its performance was stable until the maximum iteration was reached. Figure 8 shows the ROC curves of the performance of GoogLeNet on the testing data set with learning rates of 0.01, 0.001, and 0.0001.

Regarding the AlexNet network, Figure 9, Figure 10 and Figure 11 show the training process of AlexNet with the three choices for the learning rate parameter. The performance of the AlexNet also varied depending on the choice of learning rate. From Table 2, it can be observed that AlexNet achieved the highest performance when the learning rate is very small. Consequently, the sensitivity and specificity were also 100%. As Figure 10 shows, AlexNet obtained 100% accuracy at iteration 350, and its performance remained stable until the end of the training procedure. The time required for AlexNet to reach the final iteration was much less than that required by GoogLeNet, as shown in Table 3. In addition, the best CNN performance with the least time was obtained with a learning rate of 0.001. Despite the achievements of both CNNs, the learning process is not stable when using higher learning rates. As can been observed from Figure 7 and Figure 11, GoogLeNet and AlexNet show stability in the learning process with a 0.001 learning rate despite the slow convergence.

Other evaluation metrics, including the sensitivity for both CNNs, reached 100%. This means that the CNNs successfully predicted the true positive instances of the abnormality class. They also successfully predicted the true negative instances of the normality class. In addition, the study analyzed the receiver operating characteristics to test the classification performance. The curves in Figure 8 and Figure 12 demonstrate a balanced trade-off between the sensitivity and the specificity for both CNNs.

## 6. Discussion

The experiments aimed at detecting ulcers in WCE images by applying two CNN architectures, namely GoogLeNet and AlexNet. The images in this study were identical to those used in [31]. Table 1, Table 2 and Table 3 provide a summary of the performance results. The validation accuracy and losses were calculated from the last layers of AlexNet and GoogLeNet.

The CNNs were used to automatically detect ulcer regions in WCE images, obviating the need for preprocessing in order to prepare the images for classification. Table 3 summarizes the performance of the two CNN architectures in terms of runtime. It shows that AlexNet outperformed GoogLeNet by requiring 18:37 min for training, compared to 37:41 min for GoogLeNet. This translates to AlexNet requiring approximately half the time for training. This may be due to the large number of layers in GoogLeNet. However, these computational times were less when compared to traditional machine learning methods, where analysts usually spend more time and effort in understanding the images [33]. Moreover, this requires the evaluation and ranking of a large number of features, so as to choose the appropriate ones for the classification task. Feature extraction and selection are accomplished automatically using CNNs.

Based on the results, it is concluded that a high learning rate of 0.01 does not provide satisfactory results for both GoogLeNet and AlexNet. Analysis of the loss curve under the various experimental setups, as shown in Figure 7 and Figure 11, demonstrated that both CNNs achieved 100% accuracy with a learning rate of 0.0001. Moreover, as shown in Figure 10, AlexNet also provides 100% accuracy with a learning rate of 0.001. This is the best result achieved so far in ulcer image analysis, when compared to the application of state-of-the-art machine learning techniques, including deep neural networks, as shown in Table 4. We provide an extensive comparison of our results to those of traditional machine learning tools. For example, Bchir et al. [31] achieved 96% accuracy using SVMs. They attempted to extract new features by analyzing textures. In addition, Iakovidis and Koulaouzidis [48] investigated an automated way to detect lesion images, achieving 95% sensitivity. Szczypiński et al. [29] used a feature extractor based on color, which achieved 95% accuracy in both sensitivity and specificity. In all these studies, the results obtained were unsatisfactory owing to irrelevant features extracted from the WCE images that led to misclassification. Vasilakakis [49] asserted that this might be related to the significantly lower resolution of WCE images, which limits the visibility of the texture, thus affecting the amount and quality of discriminative information. More importantly, the database used in the present study contained not only instances of ulcers but also several other types of abnormalities, including for example, vascular lesions, for which texture may not be as discriminative as color [11]. In terms of the use of deep learning networks, previous studies, including Seguia et al. [35] used their designed CNN in the analysis of WCE images. Their CNN achieved 96% accuracy. Fan et al., achieved 95.16% accuracy and 96% sensitivity [10].

To further evaluate the effectiveness of the proposed methodology using CNN, we performed further simulation studies using multilayer perceptrons (MLP) with the same datasets to benchmark the proposed neural network architectures as shown in Table 5. It can be observed that the best performance achieved on the test dataset was 85% using color histograms and LBP.

However, the results of the present study showed that CNNs have the ability to automatically extract and evaluate a set of the optimal features. This is related to the numbers of layers in the CNN architecture and the fact that the present tests were designed to identify the features that help to distinguish between the two classes. To conclude, the viability of the two pretrained CNNs for ulcer detection was fully demonstrated in terms of specificity, sensitivity, accuracy, AUC, and loss. Furthermore, comparison experiments showed that the two pretrained CNNs outperform state-of- the-art methods for ulcer detection, paving the way for the development of a computer-aided diagnosis system for ulcer detection.

## 7. Conclusions

In recent years, deep learning has been at the forefront of research and technological efforts in automated analysis and recognition and has delivered significant improvements compared to traditional machine learning algorithms. Based on their performance, deep learning methods are considered by many researchers to be credible candidates for automated detection and diagnosis of abnormalities in a variety of medical images.

The novelty of this research lies in illustrating the use of pretrained CNN models for recognizing ulcer regions in WCE images. GoogLeNet and AlexNet models were pretrained on a subset of the ImageNet database to determine the best combination of network parameters that can enable these two CNNs to detect the occurrence of ulcers with high accuracy. Despite the limited number of data, both architectures demonstrated zero classification error with 100% accuracy for the identified combination of network parameter settings.

Although it was time-consuming, the experiments illustrated the excellent performances of both CNN models and demonstrated their potential in automated analysis of medical images. The promising detection rates by GoogleNet and AlexNet, in the context of the state-of-the-art results, are expected to reinforce their use in ulcer classification in WCE images. Furthermore, for the first time, an attempt was made to fine-tune pretrained CNNs for ulcer detection, which has the potential to pave the way for employing pretrained CNNs within a CAD system for accurate diagnosis.

Finally, based on the performance results of the CNNs, conclusive insights can be generalized to analyzing WCE images for other types of diseases.

## Figures and Tables

**Table 1:** Performance of GoogLeNet for training and testing data set with three different learning rates.

Performance Measurements	LR = 0.0001		LR = 0.001		LR = 0.01	
	Training Set	Testing Set	Training Set	Testing Set	Training Set	Testing Set
Accuracy	100%	100%	100%	97.143%	73.33%	76.19%
Loss	1.0093 × 10^−6^	8.6569 × 10^−8^	0.0298	0.001	2.7444	2.1683
Sensitivity	1	1	1	1	0.73	0.76
Specificity	1	1	1	1	0	0
AUC	1	1	1	0.9864	0.50	0.50

**Table 2:** Performance of AlexNet with Lr = [0.0001, 0.001, 0.01].

Performance Measurements	LR = 0.0001		LR = 0.001		LR = 0.01	
	Training Set	Testing Set	Training Set	Testing Set	Training Set	Testing Set
Accuracy	100%	100%	100%	100%	73.33%	76.19%
Loss	3.9736 × 10^−8^	3.1221 × 10^−8^	3.9736 × 10^−8^	8.5150 × 10^−8^	1.0933	3.7958
Sensitivity	1	1	1	1	0.73	0.76
Specificity	1	1	1	1	0	0
AUC	1	1	1	1	0.50	0.50

**Table 3:** Runtime costs for the learning process of the CNNs (in minutes).

Learning Rate	LR = 0.0001	LR = 0.001	LR = 0.01
AlexNet	18:37	14	08:09
GoogleNet	37:41	35:22	33:21

**Table 4:** Comparison of state-of-the-art machine learning techniques in WCE images.

References	Data Type	Classifiers	Result
[31]	Ulcer images	SVM	96% sensitivity
[35]	Small bowel	CNN	The accuracy is 96%
[10]	Ulcer images	AlexNet	96% sensitivity
[33]	Bleeding images	CNN	99% in F measure
[32]	Inflammatory gastrointestinal disease	CNN with SVM	The accuracy is 90%
[14]	Polyps images	SSAEIM	The accuracy is 98%
[50]	Ulcer images	SVM	97.68% sensitivity
[24]	Ulcer, bleeding images	SVM	98% sensitivity

**Table 5:** Simulation results for the use of multilayer perceptrons in WCE image ulcer detection.

Performance Measurements	CIE_lab Color Histogram		Local Binary Pattern		Color Coherence Vector	
	Training Set	Testing Set	Training Set	Testing Set	Training Set	Testing Set
Accuracy	83.7%	85%	85.199.6%	85%	77.3%%	76.5%
Loss	0.1530	0.1495	0.1467	0.1469	0.3115	0.3267
Sensitivity	97.5%	98.9%	99.1%	98.9%	90%	89.1%
Specificity	0%	0%	0%	0%	0%	0%
AUC	33.68%	34.67%	36.45%	32.85%	60.41%	59.96%

## References

[1] Cireşan D.C., Giusti A., Gambardella L.M., Schmidhuber J. Mitosis Detection in Breast Cancer Histology Images with Deep Neural Networks. Proceedings of the International Conference on Medical Image Computing and Computer-assisted Intervention.

[2] Deng L., Yu D. (2014). Deep learning: Methods and applications. Found. Trends^®^ Signal Process..

[3] Dong Y., Jiang Z., Shen H., Pan W., Williams L., Reddy V., Benjamin W., Bryan A. Evaluations of deep convolutional neural networks for automatic identification of malaria infected cells. Proceedings of the IEEE EMBS International Conference on Biomedical & Health Informatics (BHI).

[4] Esteva A., Kuprel B., Novoa R., Ko J., Swetter S., Blau H., Thrun S. (2017). Dermatologist-level classification of skin cancer with deep neural networks. Nature.

[5] Hoo-Chang S., Holger R., Mingchen G., Le L., Ziyue X., Isabella N., Jianhua Y., Daniel M., Ronald M.S. (2016). Deep convolutional neural networks for computer-aided detection: CNN architectures, dataset characteristics and transfer learning. IEEE Trans. Med. Imag..

[6] Khan S., Yong S.P. A Deep Learning Architecture for Classifying Medical Images of Anatomy Object. Proceedings of the Asia-Pacific Signal and Information Processing Association Annual Summit and Conference (APSIPA ASC).

[7] Wolterink J.M., Leiner T., de Vos B., van Hamersvelt R.W., Viergever M.A., Išgum I. (2016). Automatic coronary artery calcium scoring in cardiac CT angiography using paired convolutional neural networks. Med. Image Anal..

[8] Litjens G., Kooi T., Bejnordi B.E., Setio A.A.A., Ciompi F., Ghafoorian M., Van Der Laak J.A., Van Ginneken B., Sánchez C.I. (2017). A survey on deep learning in medical image analysis. Med. Image Anal..

[9] Fan S., Xu L., Fan Y., Wei K., Li L. (2018). Computer-aided detection of small intestinal ulcer and erosion in wireless capsule endoscopy images. Phys. Med. Biol.

[10] Xu Y., Mo T., Qiwei F., Zhong P., Lai M., Chang E. Deep Learning of Feature Representation with Multiple Instance Learning for Medical Image Analysis. Proceedings of the IEEE International Conference on Acoustics, Speech and Signal Processing (ICASSP).

[11] Szegedy C., Liu W., Jia Y., Sermant P., Reed S., Anguelov D., Erhan D., Vanhoucke V., Rabinovich A. Going Deeper with Convolutions. Proceedings of the IEEE Conference on Computer Vision and Pattern Recognition (CVPR).

[12] Krizhevsky A., Sutskever I., Hinton G. Image Net Classification with Deep Convolutional Neural Networks. Proceedings of the Advances in neural information processing systems.

[13] Yuan Y., Meng M. (2017). Deep learning for polyp recognition in wireless capsule endoscopy images. Med. Phys..

[14] Ronneberger O., Fischer P., Brox T. U-Net: Convolutional Networks for Biomedical Image Segmentation. Proceedings of the International Conference on Medical Image Computing and Computer-Assisted Intervention.

[15] Tajbakhsh N., Gurudu S.R., Liang J. Automatic Polyp Detection in Colonoscopy Videos Using an Ensemble of Convolutional Neural Networks. Proceedings of the IEEE 12th International Symposium on Biomedical Imaging (ISBI).

[16] Linder T., Jigin O. (2017). Organ Detection and Localization in Radiological Image Volumes. Master’s Thesis.

[17] Adler D.G., Gostout C.J. (2003). Wireless capsule endoscopy. Hosp. Physician.

[18] Fireman Z., Glukhovsky A., Jacob H., Lavy A., Lewkowicz S., Scapa E. (2002). Wireless capsule endoscopy. IMAJ-RAMAT GAN.

[19] Ojala T., Pietikäinen M. (1999). Unsupervised texture segmentation using feature distributions. Pattern Recognit..

[20] Gevers T., Smeulders A.W. (1999). Color-based object recognition. Pattern Recognit..

[21] Shafer S.A. (1985). Using color to separate reflection components. Color Res. Appl..

[22] Finlayson G.D., Hordley S.D., Tastl I. (2006). Gamut constrained illuminant estimation. Int. J. Comput. Vis..

[23] Liaqat A., Khan M.A., Shah J.H., Sharif M., Yasmin M., Fernandes S.L. (2018). Automated ulcer and bleeding classification from WCE images using multiple features fusion and selection. J. Mech. Med. Biol..

[24] Li B., Meng M. (2009). Texture analysis for ulcer detection in capsule endoscopy images. Image Vis. Comput..

[25] Charfi S., El Ansari M. (2018). Computer-aided diagnosis system for colon abnormalities detection in wireless capsule endoscopy images. Multimed. Tools Appl..

[26] Li B., Meng M. Ulcer Recognition in Capsule Endoscopy Images by Texture Features. Proceedings of the 7th World Congress on Intelligent Control and Automation, WCICA.

[27] Souaidi M., Abdelouahed A., El Ansari M. (2018). Multi-scale completed local binary patterns for ulcer detection in wireless capsule endoscopy images. Multimed. Tools Appl..

[28] Szczypiński P., Klepaczko A., Pazurek M., Daniel P. (2014). Texture and color based image segmentation and pathology detection in capsule endoscopy videos. Comput. Methods Programs Biomed..

[29] Wang C., Luo Z., Liu X., Bai J., Liao G. Detection of Protruding Lesion in Wireless Capsule Endoscopy Videos of Small Intestine. Proceedings of the SPIE Medical Imaging; Medical Imaging 2018: Computer-Aided Diagnosis.

[30] Bchir O., Ismail M., AL_Aseem N. (2018). Empirical comparison of visual descriptors for ulcer recognition in wireless capsule endoscopy video. Comput. Sci. Inf. Technol..

[31] Georgakopoulos S.V., Iakovidis D., Vasilakakis M., Plagianakos V.P., Koulaouzidis A. Weakly-Supervised Convolutional Learning for Detection of Inflammatory Gastrointestinal Lesions. Proceedings of the IEEE International Conference on Imaging Systems and Techniques (IST).

[32] Jia X., Meng M. A Deep Convolutional Neural Network for Bleeding Detection in Wireless Capsule Endoscopy Images. Proceedings of the IEEE 38th Annual International Conference on the Engineering in Medicine and Biology Society (EMBC).

[33] Wimmer G., Hegenbart S., Vécsei A., Uhl A. Convolutional Neural Network Architectures for the Automated Diagnosis of Celiac Disease. Proceedings of the International Workshop on Computer-Assisted and Robotic Endoscopy.

[34] Seguí S., Drozdzal M., Pascual G., Radeva P., Malagelada C., Azpiroz F., Vitria J. (2016). Generic feature learning for wireless capsule endoscopy analysis. Comput. Biol. Med..

[35] Pei M., Wu X., Guo Y., Fujita H. (2017). Small bowel motility assessment based on fully convolutional networks and long short-term memory. Knowl. Based Syst..

[36] Yuan Y., Wang J., Li B., Meng M. (2015). Saliency based ulcer detection for wireless capsule endoscopy diagnosis. IEEE Trans. Med. Imaging.

[37] Yeh J.-Y., Wu T.-H., Tsai W.-J. (2014). Bleeding and ulcer detection using wireless capsule endoscopy images. J. Softw. Eng. Appl..

[38] Nawarathna R., Oh J.H., Muthukudage J., Tavanapong W., Wong J., de Groen P.C., Tang S.J. (2014). Abnormal image detection in endoscopy videos using a filter bank and local binary patterns. Neurocomputing.

[39] Goodfellow I., Bengio Y., Courville A. (2016). Deep Learning.

[40] Cheng P.M., Malhi H.S. (2017). Transfer learning with convolutional neural networks for classification of abdominal ultrasound images. J. Digit. Imaging.

[41] Tajbakhsh N., Shin J., Gurudu S., Hurst R., Kendall C., Gotway M., Liang J. (2016). Convolutional neural networks for medical image analysis: Full training or fine tuning?. IEEE Trans. Med. Imag..

[42] Yosinski J., Clune J., Bengio Y., Lipson H. (2014). How Transferable are Features in Deep Neural Networks. Advances in Neural Information Processing Systems.

[43] Khan S.A., Yong S.P. (2016). An Evaluation of Convolutional Neural Nets for Medical Image Anatomy Classification. Advances in Machine Learning and Signal Processing.

[44] Sugimori H. (2018). Classification of computed tomography images in different slice positions using deep learning. J. Healthc. Eng..

[45] Alaskar H. (2018). Deep learning of EMG time frequency representations for identifying normal and aggressive action. IJCSNS Int. J. Comput. Sci. Netw. Secur..

[46] Alaskar H. (2018). Deep learning-based model architecture for time-frequency images analysis. Int. J. Adv. Comput. Sci. Appl..

[47] Dr Khoroo’s Medical Clinic/Trust. http://www.drkhuroo.in/#.

[48] Iakovidis D.K., Koulaouzidis A. (2014). Automatic lesion detection in capsule endoscopy based on color saliency: Closer to an essential adjunct for reviewing software. Gastrointest. Endosc..

[49] Vasilakakis M.D., Iakovidis D.K., Spyrou E., Koulaouzidis A. (2018). DINOSARC: Color features based on selective aggregation of chromatic image components for wireless capsule endoscopy. Comput. Math. Methods Med..

[50] Souaidi M., Abdelouahad A.A., El Ansari M. A Fully Automated Ulcer Detection System for Wireless Capsule Endoscopy Images. Proceedings of the International Conference on Advanced Technologies for Signal and Image Processing (ATSIP).

